# On Patterns of Neuropsychiatric Symptoms in Patients With COVID-19: A Systematic Review of Case Reports

**DOI:** 10.7759/cureus.25004

**Published:** 2022-05-15

**Authors:** Joyce B Idehen, Usman Kazi, Justina A Quainoo-Acquah, Bailey Sperry, Ifarah Zaman, Alireza Goodarzi, Shahzad Chida, Linette Nalbandyan, Edward W Hernandez, Vatsala Sharma, Rolanda Mulume, Oare M Okoh, Izuchukwu Okonkwo, Hailey Harrison, Oladipo T Soetan, Reema Iqbal, Marlena K Lesniowska, Ali Hussain Baloch, Ayodeji Jolayemi

**Affiliations:** 1 College of Medicine, American University of Antigua, Brooklyn, USA; 2 Psychiatry, Interfaith Medical Center, Brooklyn, USA; 3 College of Medicine, Medical University of the Americas, Nevis, KNA; 4 College of Medicine, Medical University of Lublin, Lublin, POL

**Keywords:** mental health and covid-19, psychiatry and covid-19, psychiatric symptoms of covid-19, coronavirus, covid-19

## Abstract

Coronavirus disease 2019 (COVID-19) has various neuropsychiatric manifestations, including psychotic, mood, anxiety disorders, trauma-related disorders, and cognitive disorders, such as delirium. Although the psychosocial effects of the COVID-19 pandemic contribute to an increase in psychiatric comorbidities, the COVID-19 virus is also an independent risk factor. Previous studies have revealed that the virus can invade the neural tissue, which causes an imbalance of neurotransmitters that cause neuropsychiatric symptoms. The aim of this article is to conduct a systematic review to determine the patterns of neuropsychiatric manifestations of COVID-19, discussing the frequency and its impact on pre-existing psychiatric disorders.

Thirty-nine case reports were collected and analyzed for a systematic review. They were full-text, peer-reviewed journal publications from November 2020 to February 2021. Fifty-three patients were included in our study. The most frequent symptom was abnormal/bizarre behavior (50.9%), followed by agitation/aggression (49.1%), and the third most common was altered mental status and delirium (47.2%). Only 48% of our patients had a pre-existing psychiatric disorder, including three not formally diagnosed but displayed psychiatric symptoms prior to the COVID-19 infection.

Findings suggest a positive correlation of new-onset psychiatric symptoms with the SARS-CoV-2 virus. However, the exact pathophysiology of the virus itself causing neuropsychiatric manifestations needs to be investigated further.

## Introduction and background

This paper aims to discuss the possible effects of SARS-CoV-2 on the brain and its long-term consequences in patients with neuropsychiatric and cognitive symptoms. Although multiple theories exist as to why it causes such an effect, some being due to a dysregulated immune reaction or a direct invasion of the virus into the neural tissue, the aftermath remains the same [1]. We present a systematic review of case reports and case series of neuropsychiatric manifestations of COVID-19. We discuss the nature and frequency of various neuropsychiatric symptoms. The course of these neuropsychiatric symptoms and comorbidities with other SARS-CoV-2 symptoms are discussed. The impact of having a pre-existing psychiatric condition on neuropsychiatric symptoms is also discussed.

In this review, we will use the current understanding of the pathophysiology of how SARS-CoV-2 impacts the human brain and body to further comprehend how the neuropsychiatric manifestations present. The binding of SARS-CoV-2 to the angiotensin-converting enzyme 2 (ACE2) receptor is the essential step in the pathophysiology of clinical symptoms in patients with COVID-19. It is well studied that the ACE2 receptor binds ACE and works to regulate blood pressure via the angiotensin-renin-aldosterone pathways. Once the ACE2 receptor is activated, it in return increases angiotensin II. An uncontrolled and excessive level of angiotensin II can cause massive vasoconstriction, cardiovascular disease, apoptosis, accelerated aging of cells, brain degeneration due to hypoxia, and renal failure. When SARS-CoV-2 binds to ACE2 in the respiratory and blood vessel epithelial cells, it triggers a cascade of deadly manifestations. This process can manifest alongside a cytokine storm, with increased interleukin-1, interleukin-6, and tumor necrosis factor, leading to edema, hypercoagulability, and inflammation [2]. Organ failure occurs due to inflammation that activates a massive coagulation cascade leading to medical consequences of acute respiratory distress syndrome, renal failure, hepatic failure, myocardial infarctions, and cerebral vascular accidents. In the brain, the endothelial cells are invaded via the ACE2 receptor, resulting in microglia activation. This leads to an increase in kynurenine, quinolinic acid, glutamate, and neurotransmitter depletion [3]. The imbalance of neurotransmitters and an increased neural excitation due to glutamate can cause neuronal dysfunction and loss of neurons. The presenting symptoms depend on the Brodmann area that is affected.

Patients who present to the emergency room with an active COVID-19 infection in critical condition are hospitalized for variable periods of time. During this time, they are isolated from their family, friends, and other responsibilities, leading to significant psychological stress. These patients feel immobilized and vulnerable, which increases their cortisol and steroid hormone levels - resulting in dangerous levels of cytokines [2]. These further damage various organs already at risk due to the overactive ACE2 receptors. Sustained stress can further the neuropsychiatric and neurocognitive manifestations in COVID-19 patients.

Data based on this article were previously presented as a poster board presentation at the Family Medicine Education Consortium Virtual Conference from October 8-10, 2021.

## Review

Methods

We conducted a literature review from November 2020 to February 2021 based on case reports* *amongmen and women aged 18-76 years old in order to identify studies of patients who presented with neuropsychiatric manifestations after COVID-19 infection. The MOOSE guidelines for Meta-Analysis and Systematic Reviews for Observational Studies were followed to identify study populations accurately. Literature reviews were conducted using PubMed/MEDLINE, Google Scholar, Ovid, JoVE, and ScienceDirect. The research was conducted using search terms including but not limited to “COVID-19,” ”Coronavirus,” “Psychiatric symptoms,” “Psych,” “Case report,” “Psychosis,” and “Neuropsychiatric.” These search terms were used in all possible combinations.

Case reports were included if they were published after March 2020, confirmed positive case of COVID-19, and manifested any psychiatric manifestations (i.e., altered mental status, anxiety, depression, mania, bizarre behavior). All case reports were eligible for inclusion if the patient was between 18 and 76 years old. Pediatric cases were excluded from the final analysis. Race and gender were not exclusion criteria and were included in the literature review. A history of a psychiatric illness was taken into account but did not alter the inclusion criteria. 

A total of80 case reports/case series were recorded through database searching. This resulted in a total of76case reports remaining after duplicates were removed. After careful screening, 37 case reports were not included in the review due to not being a case report, having insufficient information, a negative nasopharyngeal test for COVID-19, patients showing neurological symptoms but not psychiatric symptoms, or inability to gain access to the full text. Thirty-nine separate case reports were used, resulting in 53 patients total analyzed for the paper. To be noted, the addition of case series in our analysis can account for the reason for a higher patient volume than that of case reports. Although no statistical analysis was done to analyze the results, three authors calculated the frequencies of each psychiatric symptom.

**Figure 1:**
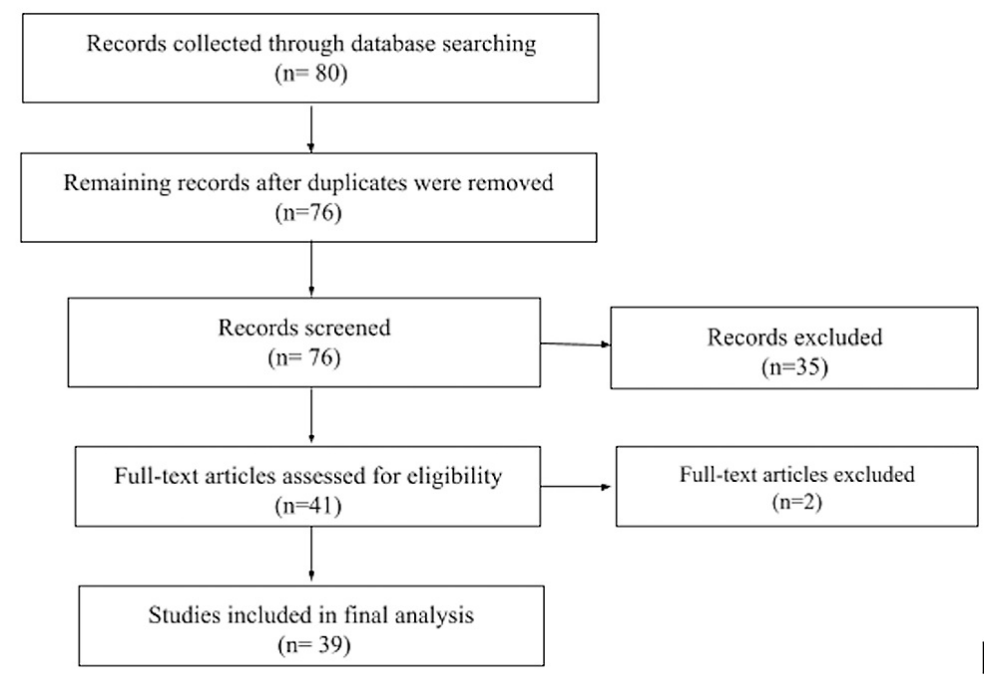
Flowchart of literature search

Case reports went through an extensive process with 12 people searching case reports, retrieving relevant information, and paraphrasing key points for analysis in excel spreadsheets. The retrieved information then went through a series of reviews each week by different reviewers for a duration of two months. Any discrepancies were addressed by all authors. There was no direct contact with any of the authors of the literature review articles. A flowchart of the* *case report search, as described before*, *is shown in Figure 1.

Results

Thirty-nine studies with a total of 53 patients were included in the final analysis [4-40].It should be noted that there were areas for potential bias from individual case reports that were analyzed*.* This includes, but is not limited to supplemental material [4,5], salary support [6], and grant support [7]. All other case reports were reviewed and showed no conflict of interest or bias. These findings did not change the outcome of our results but were acknowledged.

The demographic characteristics of patients are presented in Table 1. The majority of the cases were male patients (34/53, 64.2%) in various age groups, whereas only 19 female patients (35.8%) presented with neuropsychiatric symptoms.

**Table 1:** Demographic characteristics of the neuropsychiatric manifestations of COVID-19

	Age				
Gender	Total	18-35	36-64	65+	N/A
Male	34	6	18	6	4
Female	19	8	7	4	0

When looking at the age range of each group, the female population in the young adult range (18-35) was the most reported with eight (42.1%) cases, and the middle-age group (36-64) followed closely behind with seven (36.8%) reported cases and the elderly female population (65+) with only four (21%) reported cases. This would suggest that the young adult group (18-34) presented the most neuropsychiatric manifestations in the female population. The male population, however, had the highest reports in the middle-age group (36-64) with 18 (52.9%) reported cases, with only six (17.6%) reported cases in both the young adult (18-35) and elderly (65+); however, four cases in the male population had unreported ages, leaving an inconclusive gap (11.4%) for the male population.

Dividing the age groups up into young adults (18-35), middle-age (36-64), and elderly (65+) for both males and females and comparing them to the overall population studied showed different results. The young adult group (18-35) had a total of 14 (26.4%) reported cases, the middle-age group (36-64) had a total of 25 (47.2%) reported cases and the elderly group (65+) had a total of 10 (18.9%) reported cases, leaving the inconclusive group at a total of four (7.5%) reported cases. This study would suggest that the middle-aged (36-64) group presented more frequently with neuropsychiatric manifestations, followed by young adults (18-34) and the elderly (65+).

Majority of the patient population presented at the emergency department with fever, dyspnea, or cough, although some presented without any respiratory symptoms and solely psychiatric symptoms. Some patients did present in an outpatient setting. All cases were confirmed with a positive nasopharyngeal swab. 

**Figure 2:**
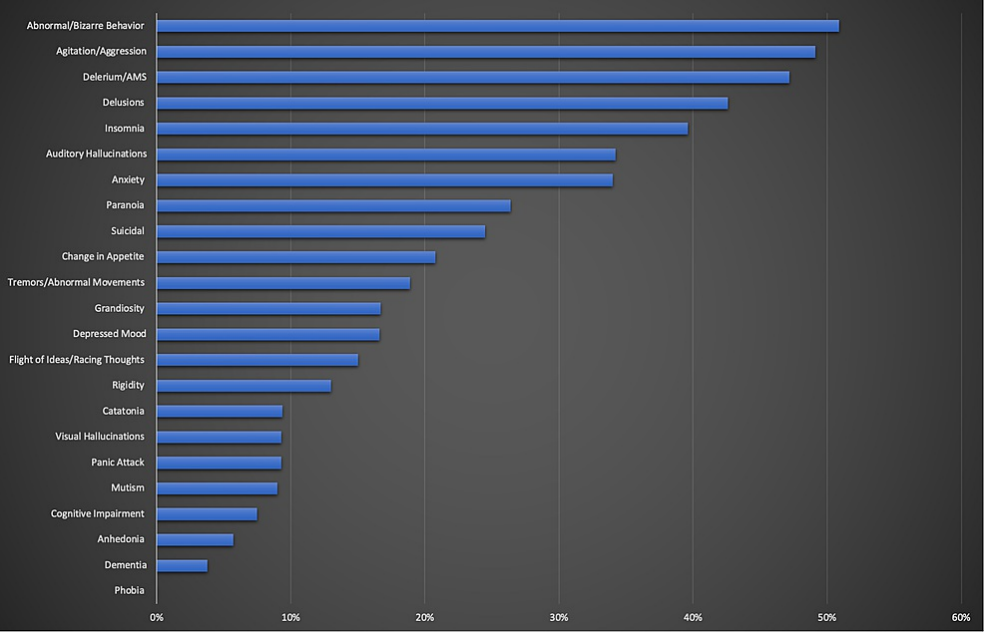
The frequency of psychiatric manifestations of COVID-19

The frequency of psychiatric manifestations in the patient population is displayed in Table 2 and Figure 2. The most frequent symptom was abnormal or bizarre behavior (50.9%), and the second most was agitation or aggression (49.1%). The third most common symptom was altered mental status and delirium (47.2%). It was interesting to observe that 43.4% of the patients had experienced delusions, while 39.6% experienced insomnia. The patients who had experienced anxiety or auditory hallucinations were equal as they both included 34% of the patients. The frequency of patients experiencing suicidal thoughts was 24.5%, which is almost tied with the frequency of patients who had symptoms of paranoia (26.4%). Tremors and abnormal movements were seen in 18.9% of patients. Surprisingly depression and grandiosity manifested equally amongst patients, with only 17% each. Flight of ideas and racing thoughts were seen in 15.1% of the patients. The rigidity of limbs was experienced in 13.2% of the patients, and 20.8% had a change in appetite. Catatonia was seen in just over 9.4% of patients. Interestingly enough, mutism, visual hallucinations, and panic attacks were all seen in 9.4% of patients. Cognitive impairment was seen in 7.5% of patients along with COVID-19 symptoms. Anhedonia and dementia at 5.7% and 3.8%, respectively, were the least seen symptoms among patients. There were no cases of phobia observed in the COVID-19 patients. 

**Table 2:** Neuropsychiatric manifestations of COVID-19

Clinical Presentation	N	%
Depressed Mood	9	17%
Change in Appetite	11	20.8%
Anxiety	18	34%
Anhedonia	3	5.7%
Delirium/AMS	25	47.2%
Dementia	2	3.8%
Suicidal thoughts	13	24.5%
Panic Attack	5	9.4%
Phobia	0	0%
Abnormal/bizarre thoughts	27	50.9%
Delusions	23	43.4%
Paranoia	14	26.4%
Auditory Hallucinations	18	34%
Visual Hallucinations	5	9.4%
Grandiosity	9	17%
Flight of ideas/racing thoughts	8	15.1%
Insomnia	21	39.6%
Catatonia	5	9.4%
Mutism	5	9.4%
Agitation/Aggression	26	49.1%
Tremors/Abnormal Movement	10	18.9%
Rigidity	7	13.2%
Cognitive Impairment	4	7.5%

We observe the timing of psychiatric symptoms to determine if it was first observed before or after a confirmed COVID-19 infection. The results showed that 60.3% of cases had developed respiratory symptoms of COVID-19 before any psychiatric symptoms. The number of days the patients presented with psychiatric symptoms ranged from 1 to 173 days. Only 18.8% of patients had severe COVID-19 that required critical care and intubation. Approximately half of the patient population (48%) had a pre-existing psychiatric disorder, including three who were not formally diagnosed but displayed psychiatric symptoms prior to COVID-19 infection. A previous history of opioid abuse was seen in 0.05 % of the patients, equal to the number of patients who had a history of alcohol abuse. Similarly, 0.04% of patients had a history of marijuana use. 

Only 30% of the patients were admitted to the inpatient psychiatric floor, while haloperidol was administered to 40% of the patients for treatment. Olanzapine was the second most common medication administered to the patients (32%). Lorazepam was given to 26% of the patients, and 13% of the patients were treated with Valproic Acid. Lastly, Chlorpromazine was only used for 0.05% of patients.

Discussion

The psychosocial impact of COVID-19 worldwide mainly revolves around the effects of the pandemic itself (isolation, economic downfall, death of loved ones, etc.). However, there are additional clinical correlations that can be made between SARS-CoV-2 and psychiatric symptoms. We highlighted the presence of psychiatric manifestations in patients who contracted the virus but had no prior history of any psychiatric conditions or exacerbations of their psychiatric disorder. The expected psychiatric symptoms of mood disorders, such as major depressive disorder, generalized anxiety disorder, can be seen. Still, interestingly, they are not seen as the majority of these psychiatric cases. We observed that the most common psychiatric symptom in patients with SARS-CoV-2 virus is a new onset symptom of bizarre behavior. A discrepancy can be made with the definition of that symptom and raise the question of “what is defined as bizarre or abnormal behavior?” To which we answer that it is new behaviors present in patients after recovering from SARS-CoV-2 that are not typically seen, such as eccentric language, misinterpretation of reality, etc. Agitation or aggression is also present as one of the most frequently defined psychiatric symptoms, which is an astonishing finding since it is more prominent than depression or anxiety. We also illustrate the demographic shifts of symptoms by gender, to which the virus created an onset of new psychiatric symptoms in more males than females. The young adult age groups were affected more in females, and males, and the middle adult age groups were affected more. Middle-aged males overall presented with the most amount of cases of new-onset of psychiatric symptoms.

We only included case reports and case series in our study, thus limiting us to draw a conclusion of having a past psychiatric illness is a risk factor for exhibiting psychiatric manifestations of COVID-19. We also had a small sample size of 53 patients. Our study could have improved if there was information on follow-up care to determine the longevity of the psychiatric manifestations and if symptoms dissipated as the infection resolved. Further details of socio-economic factors could have potentially been confounding factors or independent stressors that could have led to psychiatric symptoms of SARS-CoV-2.

Although our research presents a positive correlation of new-onset psychiatric symptoms with the SARS-CoV-2 virus, there is still some uncertainty as to the exact pathophysiology mechanism that causes this to be. Future research can explore this topic in more detail, on how specifically the virus itself causes a hormonal imbalance in the brain or other potential causes. A good thing to note is that the majority of these cases were treated with Haloperidol and other antipsychotic drugs as well as mood stabilizers, showing an association between hormonal shifts in the brain from the viral infection and treatment management.

## Conclusions

The goal of this article was to conduct a systematic review to determine patterns of neuropsychiatric manifestations in patients with COVID-19, and to discuss their frequency and impact on pre-existing psychiatric disorders. Thirty-nine case reports were collected and analyzed for systematic review, and 53 patients were included in the study. The most frequent symptom found was abnormal or bizarre thoughts and behavior (50.9%). The second most common finding was agitation or aggression (49.1%). The third most common reported symptom was delirium or altered mental status (47.2%). These three sets of symptoms can theoretically occur with most psychiatric and medical disorders, but the rest of the reported symptoms can generally be placed into different symptom categories, including psychotic symptoms, symptoms of depression, bipolar-like symptoms, symptoms of anxiety, Parkinsonism, and dementia-like symptoms. In looking at purely psychotic symptoms, 23 patients exhibited delusions (43.4%); 18 expressed auditory hallucinations (34%); 14 displayed paranoia (26.4%); five experienced visual hallucinations (9.4%); five experienced catatonia (9.4%); and five displayed mutism (9.4%). In examining symptoms of depression, only nine reported depressed mood (17%); three reported anhedonia (5.7%); 21 experienced insomnia (39.6%); 13 experienced suicidal ideations (24.5%); and 11 described changes in appetite (20.8%). In exploring bipolar symptoms, the previous symptoms described for depression can be used for the depressive phase of bipolar disorder, but in addition, nine patients described experiencing grandiosity (17%) and eight patients experienced racing thoughts (15.1%). In looking at anxiety symptoms, 18 patients described feelings of anxiety (34%); five experienced panic attacks (9.4%); and zero experienced phobia (0%). In looking at symptoms of Parkinsonism, 10 patients described tremors or abnormal movements (18.9%), while seven experienced rigidity (13.2%). In looking at dementia-like symptoms, two patients endorsed having dementia (3.8%), and four described cognitive impairment (7.5%).

Once again, it is important to note that 52% of our sample population had no prior history of psychiatric illness or had never experienced an exacerbation of a pre-existing psychiatric illness in the past. While the exact pathophysiological mechanism of how COVID-19 exerts its influence on the central nervous system is yet to be discovered, the fact that there is such a wide array of neuropsychiatric symptoms, many of which are familiar to us from the more prevalent psychiatric disorders, suggests a correlation between COVID-19 and these neuropsychiatric manifestations, and that the exact mechanism may be quite complicated. For example, the dopaminergic theory of schizophrenia is based on the high dopaminergic tone in the mesolimbic pathway, and the low dopaminergic tone in the mesocortical pathway that causes the negative symptoms of schizophrenia. Low serotonin is believed to be the cause of symptoms of major depressive disorder. Yet all of these neurotransmitters and pathways are being affected by COVID-19. Further research will be required to fully understand the neuropsychiatric manifestations brought upon by COVID-19, specifically, longitudinal studies that can follow this unique patient population that is experiencing neuropsychiatric symptoms for the first time. Only 30% of the patient population in this study were admitted to an inpatient psychiatric unit, perhaps due to the overburdening of the health care system last year where there were periods when only the most dire COVID-19 cases could be admitted. Following up with these patients could determine whether these neuropsychiatric manifestations occurred as a single event, or if they are long-term sequelae of COVID-19 where these patients will continue to suffer from these symptoms on a long-term basis. This research should also focus on which treatment options are effective. For example, if treating the underlying disorder is successful in resolving these neuropsychiatric symptoms effectively, then there may not be a need to start these patients on conventional psychotropic medications and expose them to potential side effects.
